# Supplementary material for: Histone H3.3 sub-variant H3mm7 is required for normal skeletal muscle regeneration
Source: Nat Commun. 2018 Apr 11;9:1400. doi: 10.1038/s41467-018-03845-1 (PMC5895627; doi:10.1038/s41467-018-03845-1)
Supplement: Supplementary file 1 — Supplementary Information [file 41467_2018_3845_MOESM1_ESM.pdf]

Histone H3.3 sub-variant H3mm7 is required for normal skeletal muscle regeneration

Harada, Maehara et al.

Supplementary Information

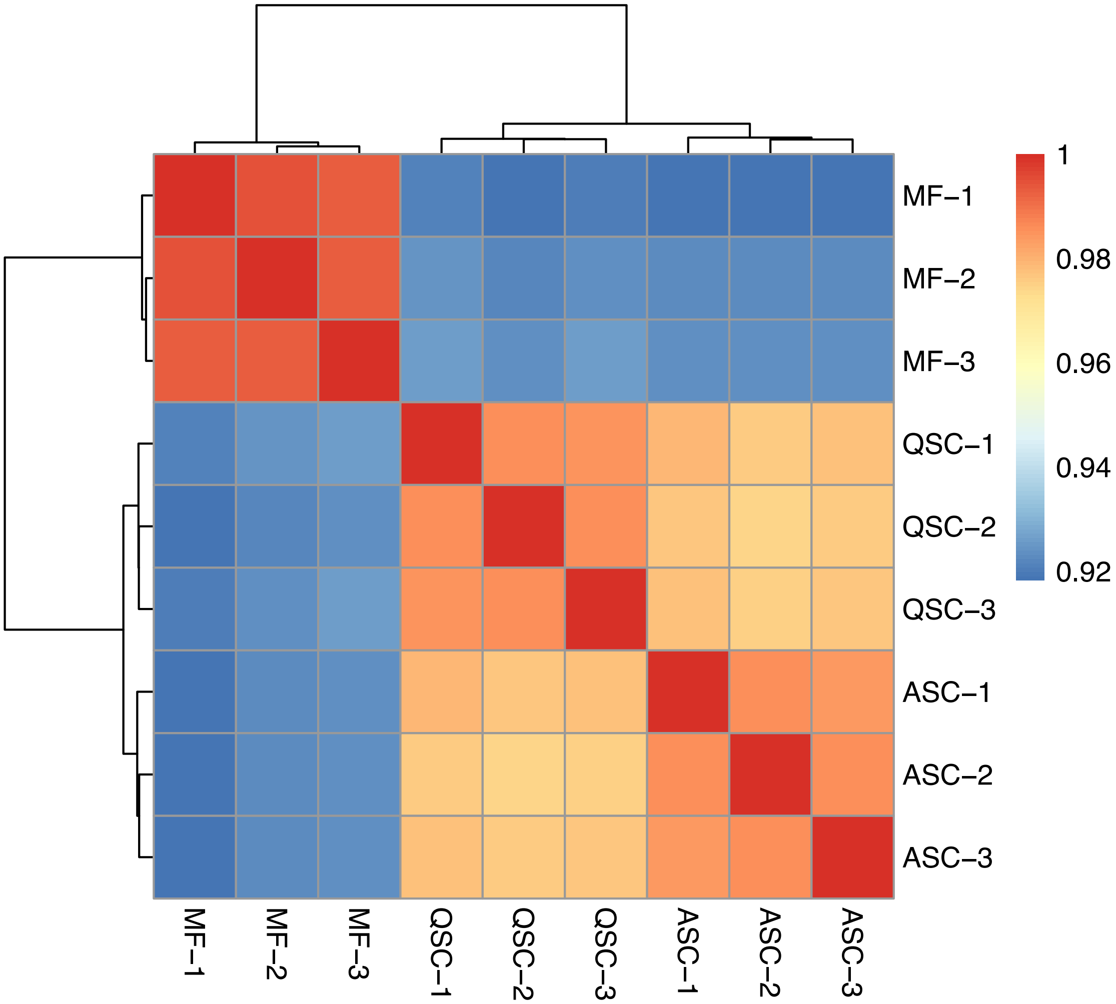

Supplementary Figure 1 | Reproducible quantification of gene expression in the skeletal muscle tissue sample using CEL-Seq2. The colors indicate the Pearson correlation coefficients of gene expression profiles. The abbreviations are QSC (quiescent satellite cells), ASC (activated satellite cells) and MF (myofiber) and the numbers (−1, 2, 3) indicate the index of the replicates.

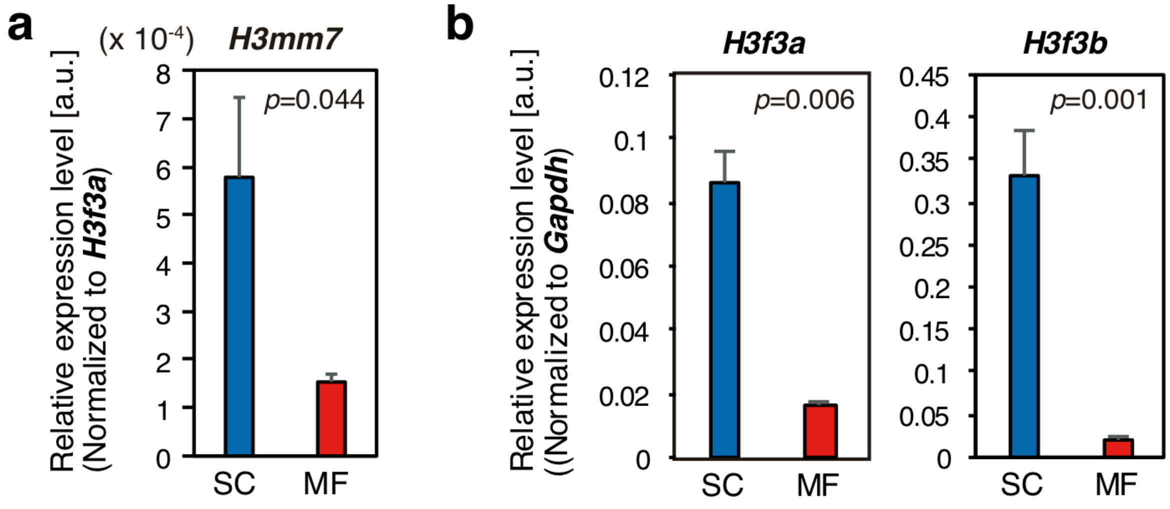

Supplementary Figure 2 | *H3mm7* is expressed in satellite cells. The RNA expression levels of *H3mm7* (allele specific PCR), *H3f3a* and *H3f3b* (RT-qPCR) in satellite cells and myofibers are shown (n=3). The relative expression level to *H3f3a* (**a**) and *Gapdh* (**b**). The abbreviations are SC (satellite cell) and MF (myofiber). Two-sided Welch's t-test was performed (n=3).

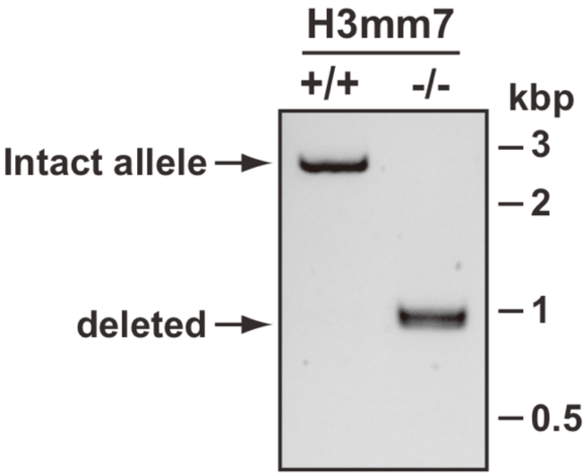

Supplementary Figure 3 | Agarose gel electrophoresis pattern of mouse genotyping. H3mm7+/+ and H3mm7-/- represent the PCR products from the wild-type (Intact) and knockout alleles (deleted), respectively.

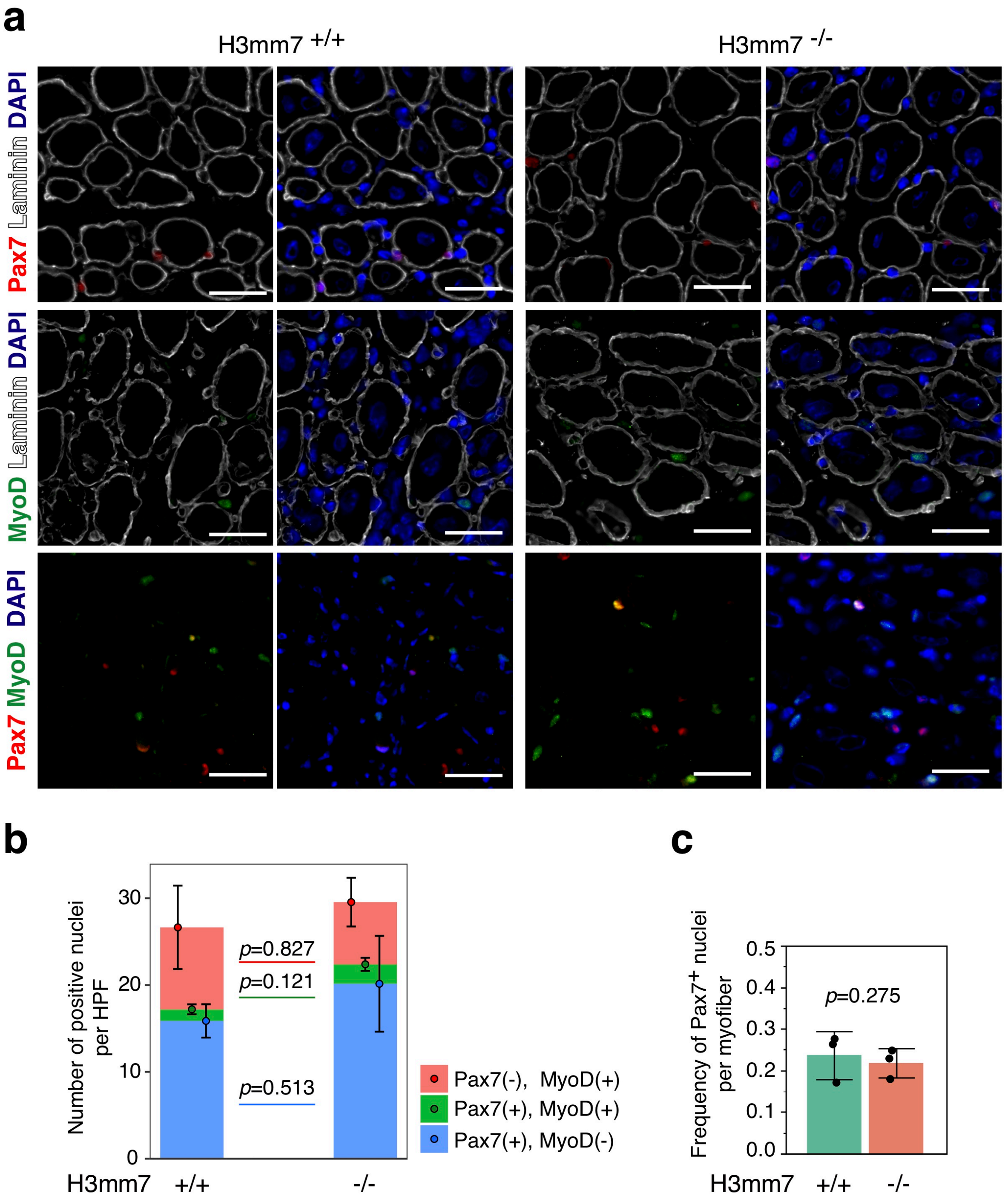

Supplementary Figure 4 | H3mm7 knockout did not affect the number of satellite cells at day five after CTX injury. **(a)** Representative images of immunohistochemistry. The bars are

25 μm. **(b)** Populations of Pax7(+)/MyoD(-), Pax7(+)/MyoD(+), and Pax7(-)/MyoD(+) cells out of total Pax7(+) and/or MyoD(+) cells are plotted (>377 cells for each mouse). For each population, no significant difference was obtained between wild-type and knockout mice. **(c)** Frequency of Pax7(+) cells per regenerated myofiber containing a central nucleus is plotted (>1000 myofibers for each mouse). For each population, no significant difference (Wilcoxon signed-rank test) was obtained between wild-type and knockout mice.

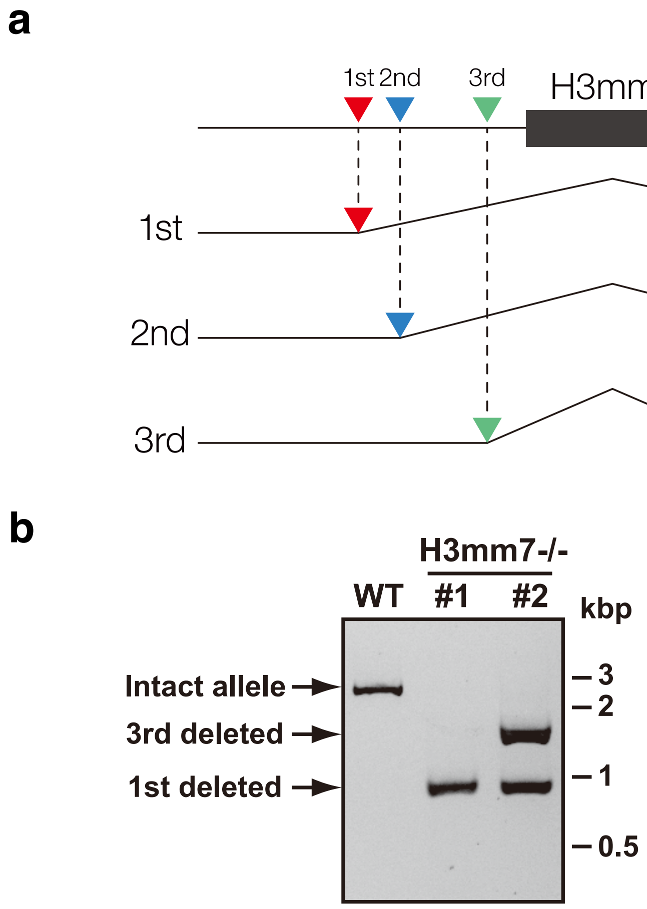

Supplementary Figure 5 | (a) Strategy for constructing H3mm7 knockout C2C12 cells using the three-step deletion of the *H3mm7* locus using the CRISPR/Cas9 system. The guide RNA was designed around the *H3mm7* locus. **(b)** The electrophoresis pattern of genotyping on agarose gels. WT and *H3mm7*$^{-/-}$ represent the PCR products from the wild-type (Intact) and knockout alleles (1st and 3rd), respectively.

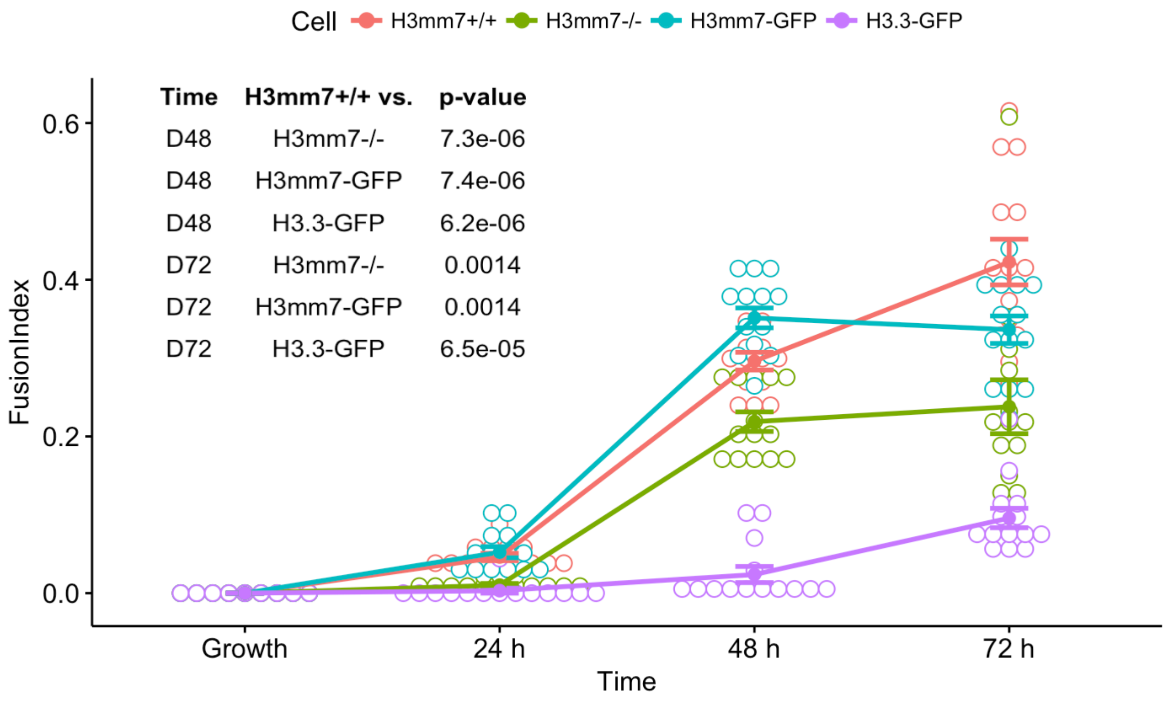

Supplementary Figure 6 | *H3mm7*$^{-/-}$ resulted in a reduction of multinucleated myofiber formation. Each dot indicates the average fusion index with the standard errors of H3mm7$^{+/+}$ (red), H3mm7$^{-/-}$ (green) and rescued cells expressing H3mm7 (blue) and H3.3 (purple), respectively. The circles indicate each measurement. The Wilcoxon signed-rank test was performed to compare against the fusion-index of H3mm7$^{+/+}$ at each time-point. The results are shown at upper-left (n=14 and p < 0.01 for every pair).

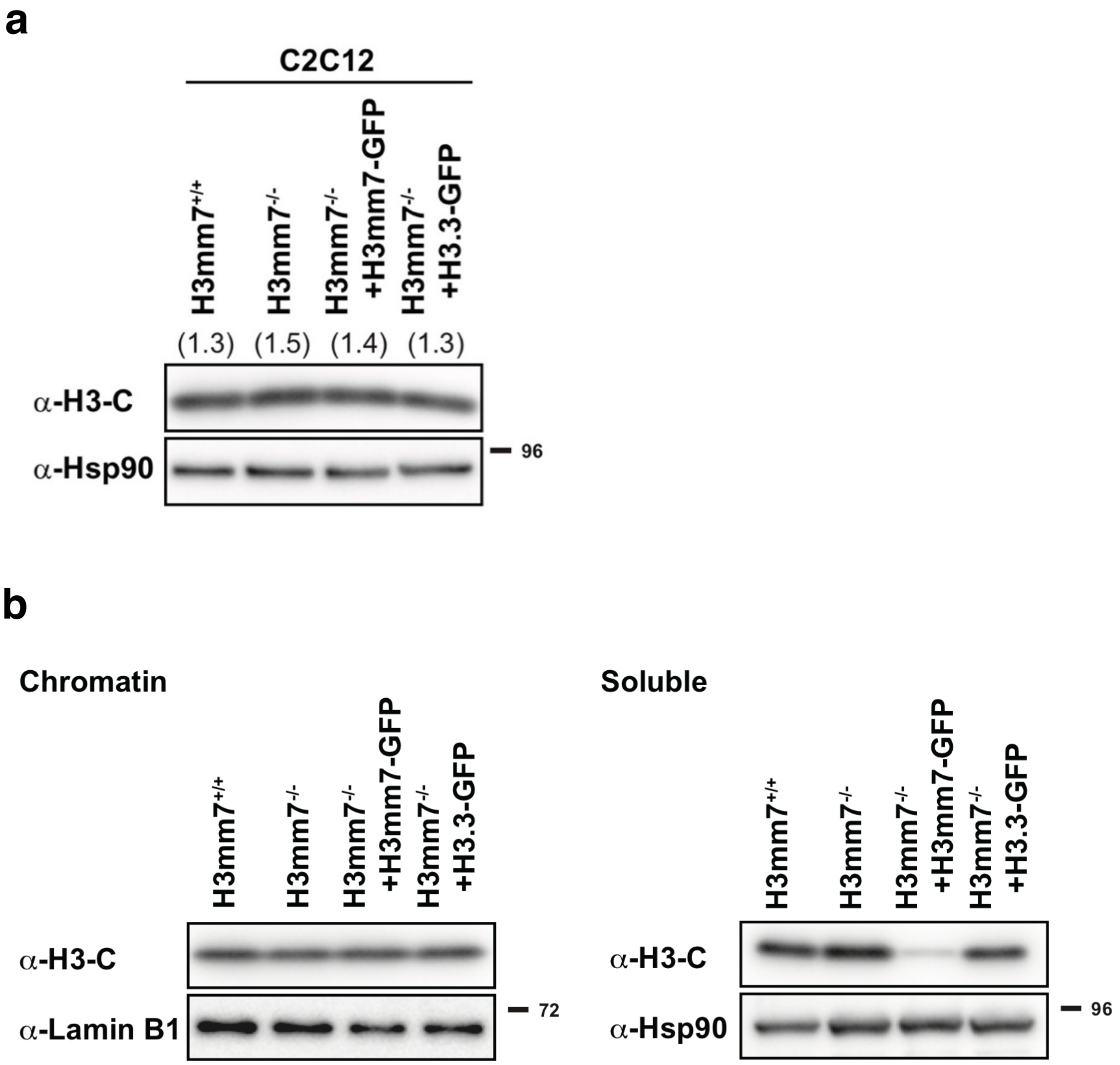

Supplementary Figure 7 | The *H3mm7* deletion and rescued cells with over-expressed GFP-fused H3 variants in C2C12 cells did not affect the total amount of endogenous H3. (**a**) Immunoblotting was performed to analyze whole H3 levels. Hsp90 levels were monitored as a loading control. The band intensities in parentheses are the ratios of the total H3 level to the level of Hsp90. (**b**) Total H3 levels in chromatin and soluble fractions. Lamin B1 levels were used as nuclear loading control.

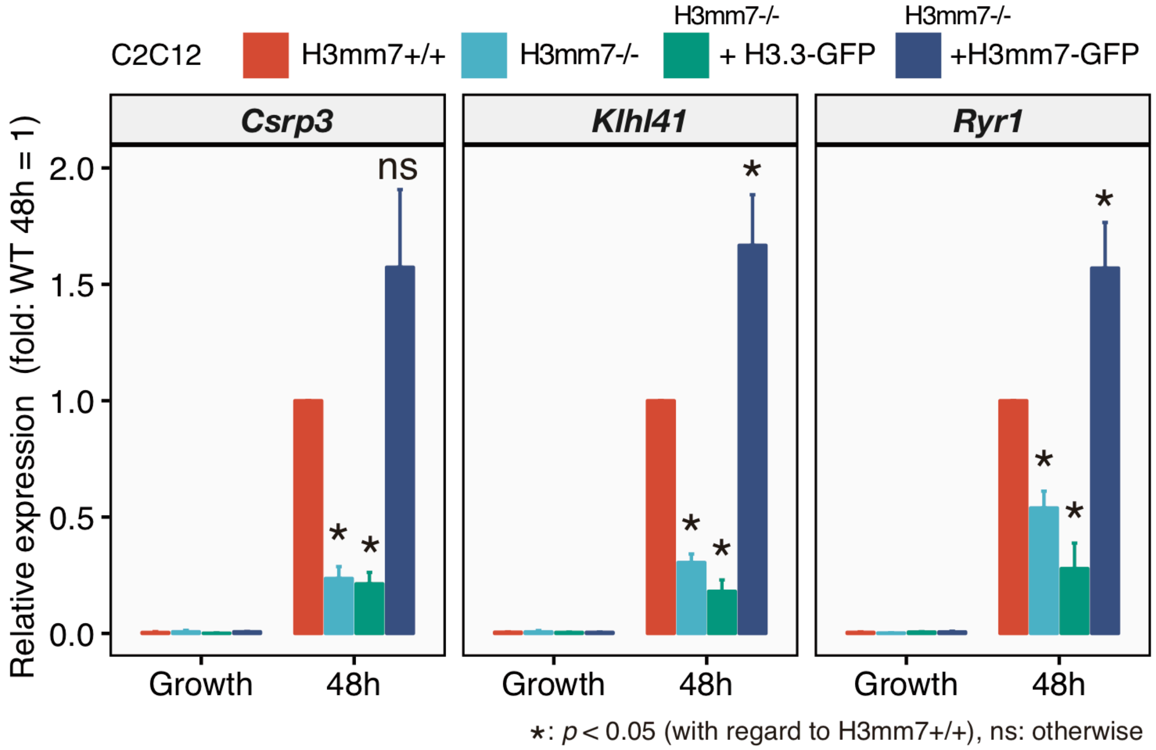

Supplementary Figure 8 | H3mm7 knockout decreased the gene expression levels of skeletal muscle components during differentiation. The expression levels relative to H3mm7$^{+/+}$ C2C12 cells at 48 h after differentiation stimuli were measured by RT qPCR. The error bars are ± 1 SD. All tests were performed with regard to H3mm7$^{+/+}$ at 48h using two-sided Welch's *t*-test (*n*=3).

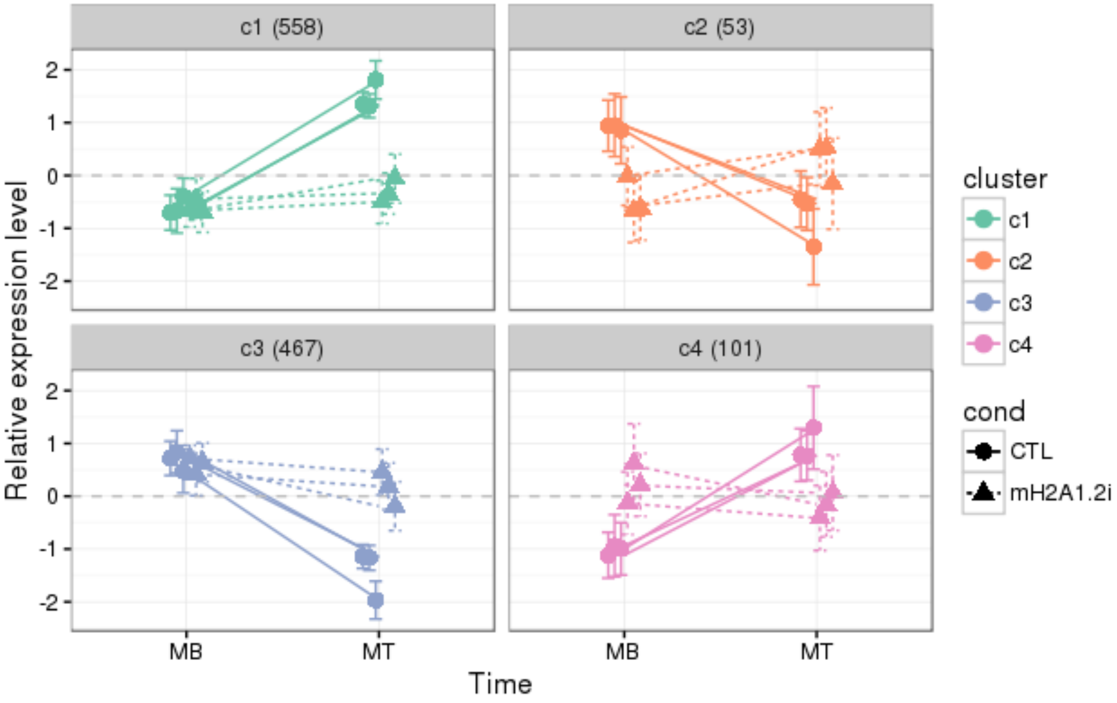

Supplementary Figure 9 | DEGs with interaction terms in si-mH2A1.2 in C2C12 cells. The four clusters of non-parallel patterns were extracted using the published RNA-seq data [NCBI SRA: SRP067391]. The data were analyzed as in Figure 2a, b (see RNA-seq data analysis section in Materials & Methods). The numbers of the genes in the clusters are indicated in parentheses. Abbreviations: MB = myoblast, MT = myotube, cond = cell condition (cell-type), CTL = control C2C12 cells, mH2A1.2i = MacroH2A1.2 siRNA-transfected C2C12 cells.

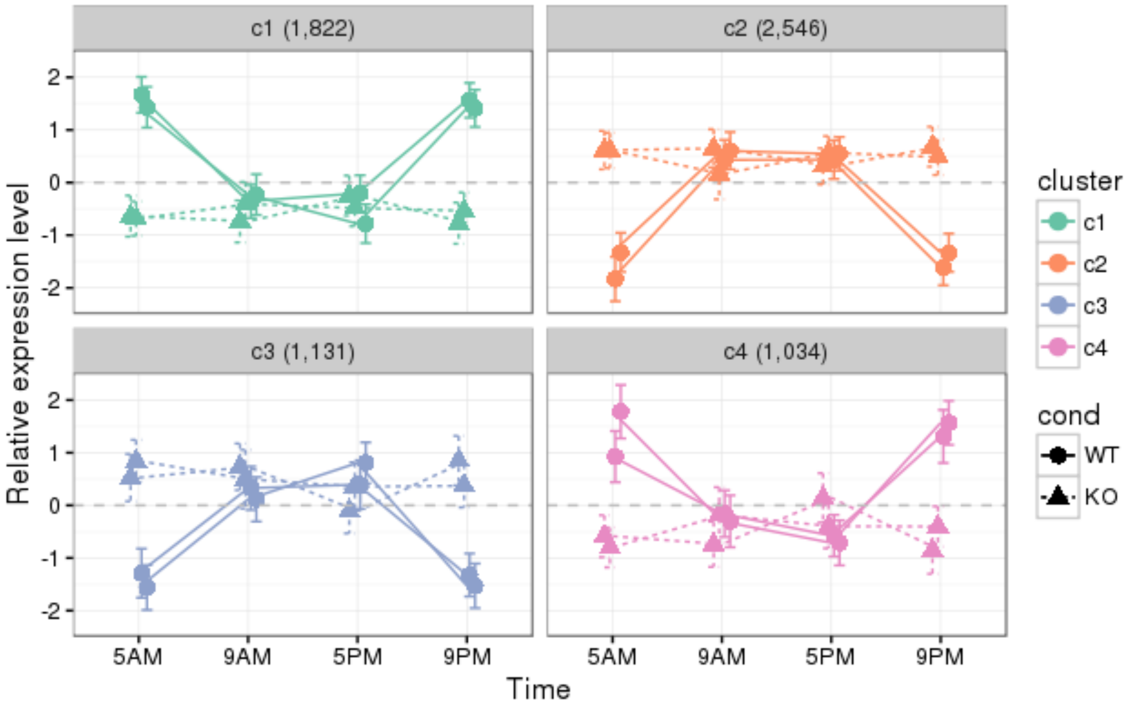

Supplementary Figure 10 | DEGs with interaction terms in a mouse hip cartilage sample of *Bmal1* knockout mice [NCBI SRA: ERP009958]. The data analysis was performed as shown above. Abbreviations: cond = cell condition (cell-type), CTL = control, WT = wild-type mice, KO = *Bmal1* knockout mice.

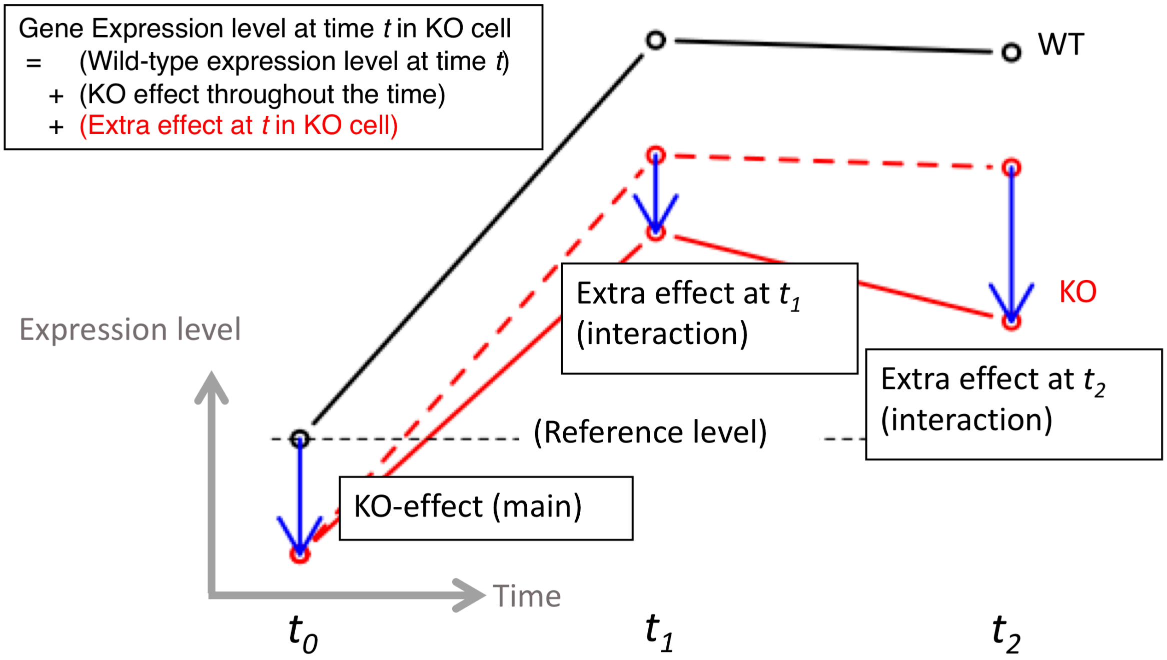

Supplementary Figure 11 | Illustration of interaction terms in a statistical linear model. Any genes expression pattern in the three time points and two conditions (arbitrary six points in coordinates of time and expression level) can be equivalently represented as the sum of six individual *effect* terms: the reference levels (baseline; the expression level of WT at t_0), the time effect at t_1 and t_2 (the differences from reference level), the main effect (an offset from the reference in KO condition) and two interaction terms (the extra effect) at t_1 and t_2. Note that the time-course pattern of KO becomes parallel to WT if there are no interaction terms (i.e., there is only the main effect; the dotted line).

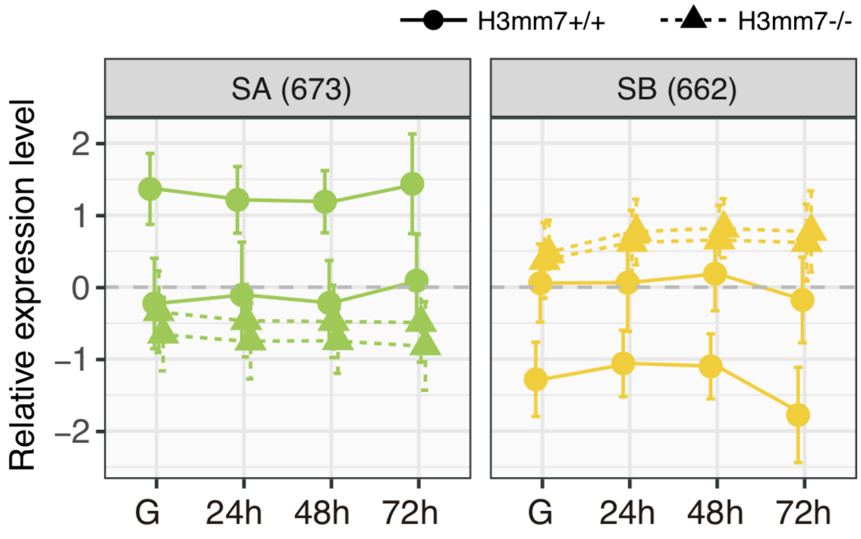

Supplementary Figure 12 | Patterns of the variation in the basal expression level of housekeeping genes in the two independent clones of *H3mm7*$^{+/+}$ C2C12 cells were extracted as clusters SA and SB in addition to the clusters in Figure 2b. Because the basal levels were different in the replicates of *H3mm7*$^{+/+}$, DESeq2 captured the difference in average expression level between *H3mm7*$^{-/-}$ and *H3mm7*$^{+/+}$.

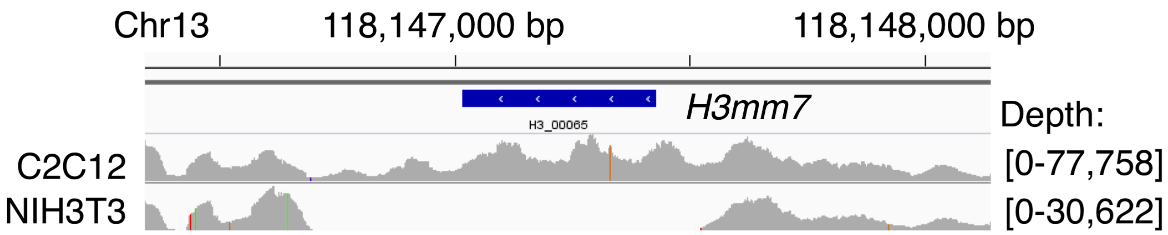

Supplementary Figure 13 | NIH3T3 cells naturally lack the *H3mm7* locus. The IGV snapshot shows the depths of genomic reads from input DNA control samples (amplicon sequence) around the *H3mm7* gene locus. The input DNA control sample of C2C12 cells is shown as a control.

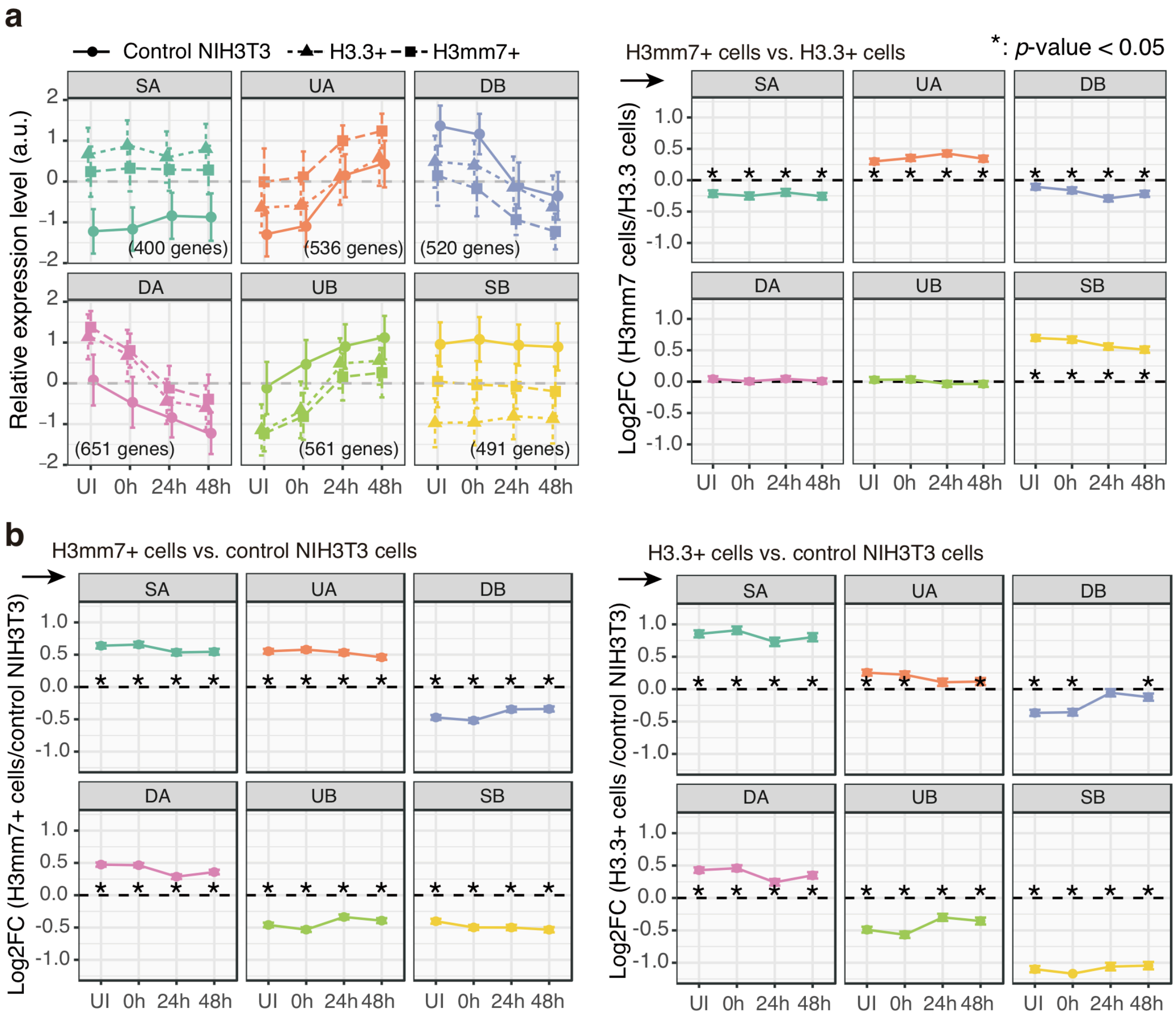

Supplementary Figure 14 | H3mm7 markedly enhanced the up-regulation (UA) and down-regulation (DB) of genes during differentiation. **(a)** The differences between the average relative expression levels of H3.3+ and H3mm7+ cells at each time point are shown. The left panel from Figure 3b was added here for clarification. **(b)** The differences between the average relative expression levels of H3.3+ (left) vs. control, and H3mm7+ vs. control NIH3T3 cells (right) at each time point. The error bars are 95% confidence intervals of the differences. The asterisk marks the p-value < 0.05 (null hypothesis: the difference is 0) of a two-sided t-test after Bonferroni correction for multiple comparisons.

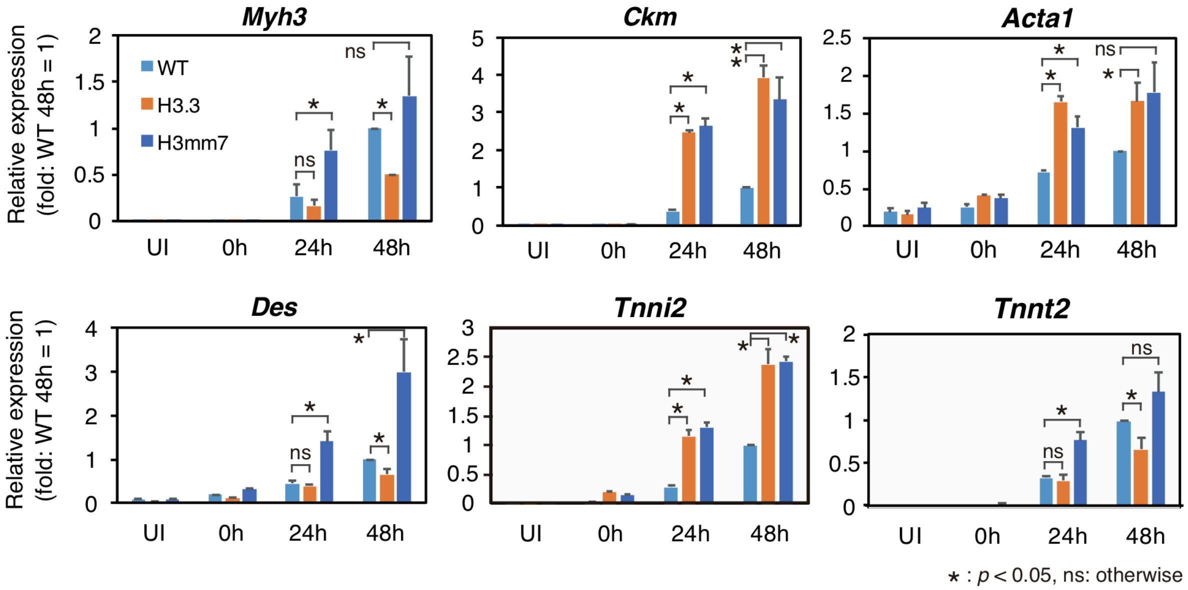

Supplementary Figure 15 | H3mm7 enhanced the upregulation of skeletal muscle markers during differentiation in NIH3T3 cells. The expression levels relative to WT cells at 48 h after differentiation stimuli were measured by RT qPCR. The error bars are ± 1 SD. Two-sided Welch's *t*-test was performed (*n*=3).

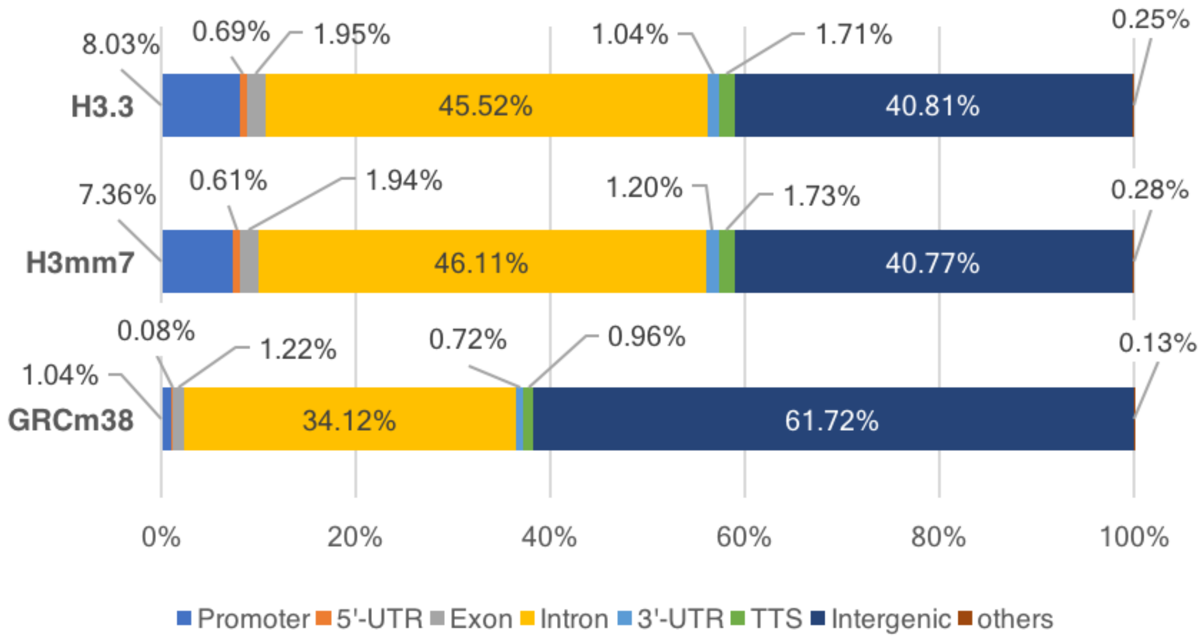

Supplementary Figure 16 | H3.3 and H3mm7 peaks show analogous genomic feature distributions in comparison with the whole genome. The GFP ChIP-seq peaks were derived (H3.3: 21,852 peaks, H3mm7: 19,463 peaks) with BCP software (version 1.1) by running the command BCP_HM, with the options -f 200 -w 200 -p 0.001. These were annotated using HOMER (version 4.8) by running annotatePeaks.pl with the option -annStats. The third bar "GRCm38" is added as a control indicating the genomic distribution of the whole HOMER annotations. The promoter enrichment of peaks was evaluated using the binomial test (p = 10^{-918} for H3.3 and 10^{-702} for H3mm7 compared to the random distribution of peaks in the reference genome GRCm38).

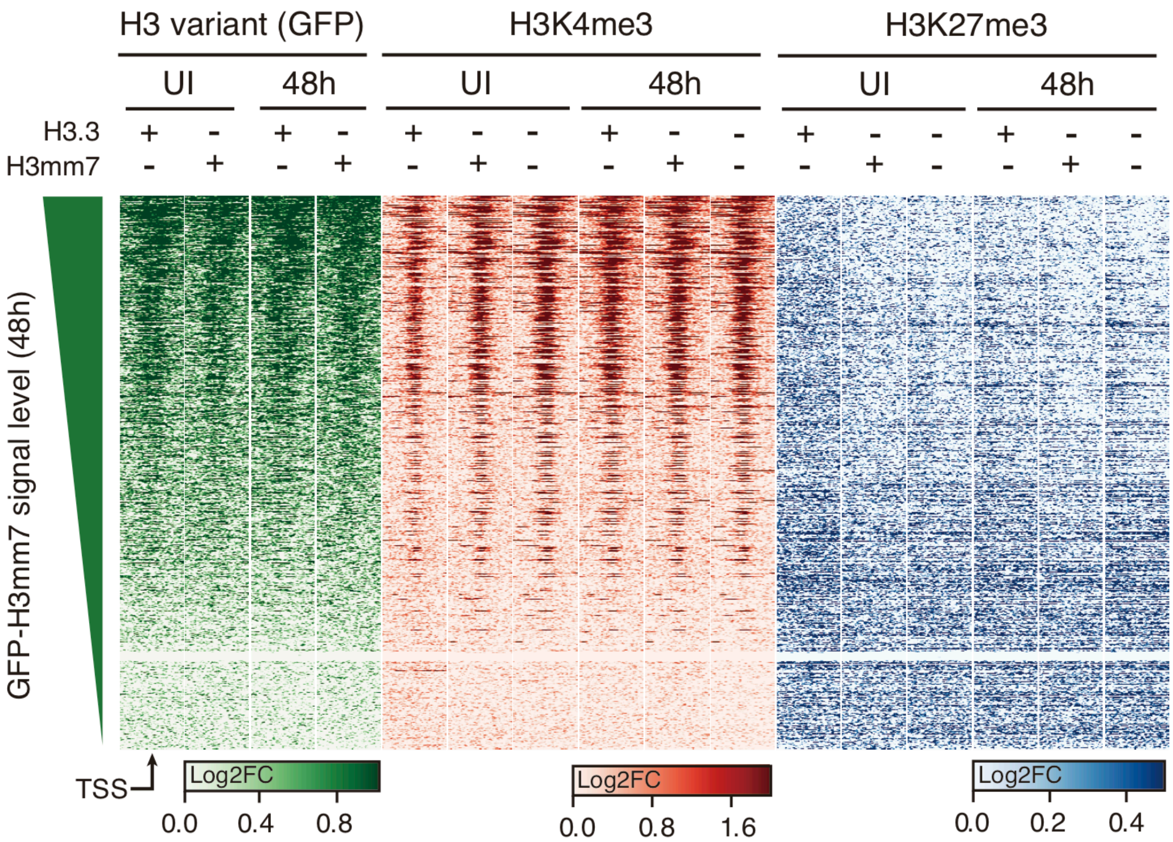

Supplementary Figure 17 | Both GFP-H3.3 and GFP-H3mm7 signal levels were positively correlated to active marks and negatively correlated to repressive marks of histone modification. The heatmap shows the ChIP-seq signal levels (log2FC of ChIP/Input signals) on gene promoters (TSS ± 5 kb). The genes (rows) were ordered by GFP-H3mm7 signal levels.

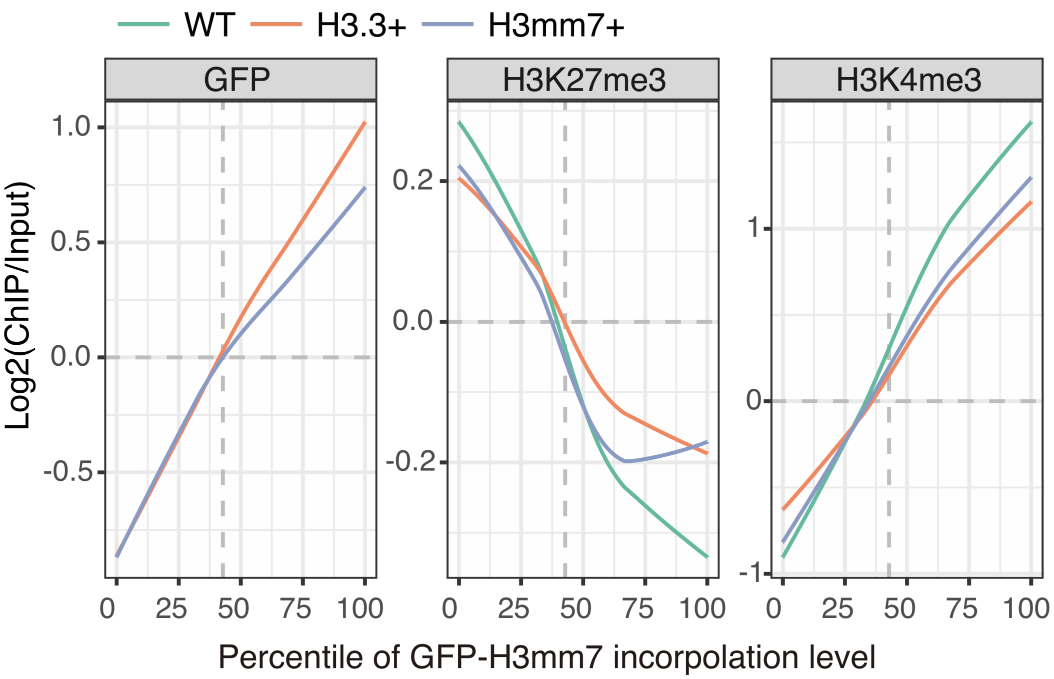

Supplementary Figure 18 | Low impact of H3mm7 incorporation on histone modifications. The average ChIP-seq signal levels in each condition (WT/H3.3+/H3mm7+) were ordered by GFP-H3mm7 signal level at 48 h after MyoD infection. The log2 fold-change of ChIP-seq signal was calculated as log2(ChIP/Input) after the median of ratio normalization of ChIP and the corresponding input sample.

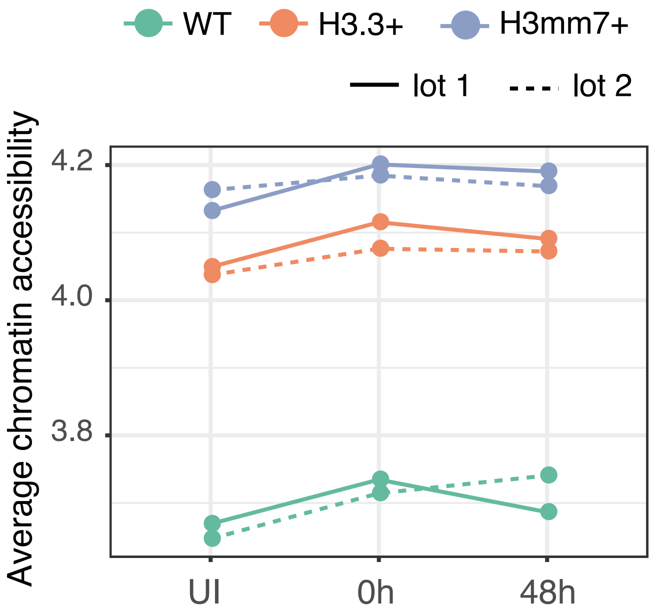

Supplementary Figure 19 | Greater chromatin accessibility was acquired by H3mm7 incorporation. The average chromatin accessibility levels of the differentially accessible genes (H3mm7+ vs. control NIH3T3 cells) are shown at each time point. Replicates (lot2) are shown as dotted lines.

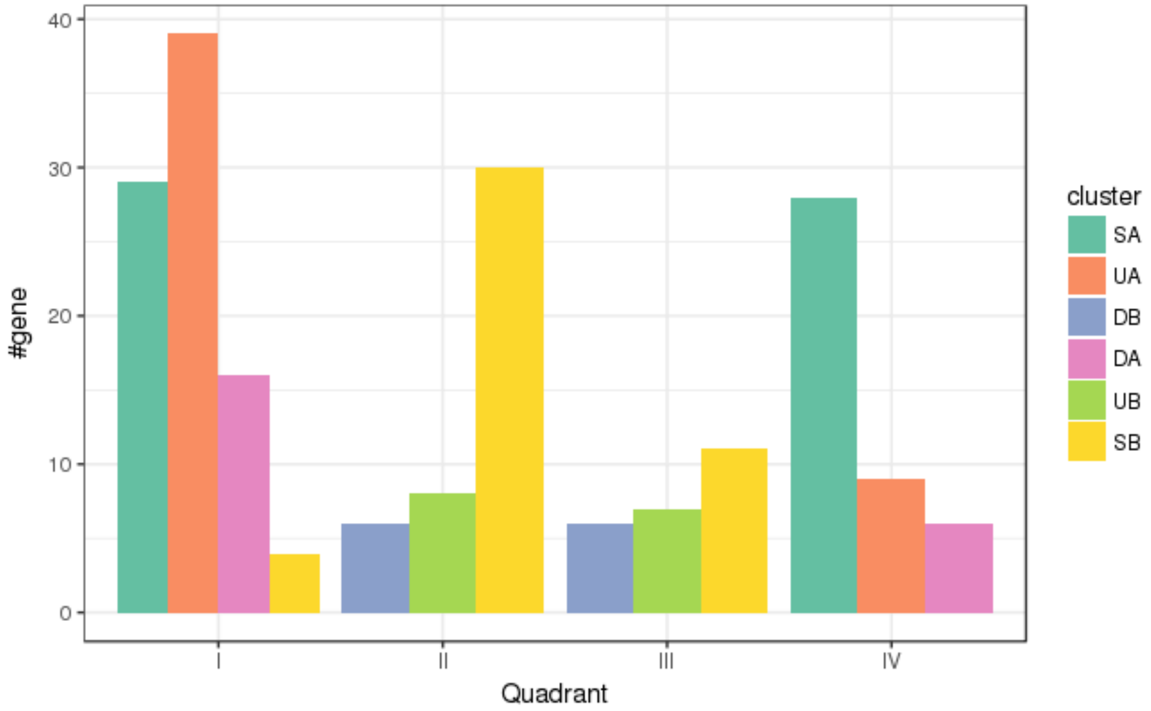

Supplementary Figure 20 | The number of genes in cluster UA were preferentially located in the I-th quadrant. The heights of the bars indicate the number of genes (points) that are located in each quadrant in Figure 3c.

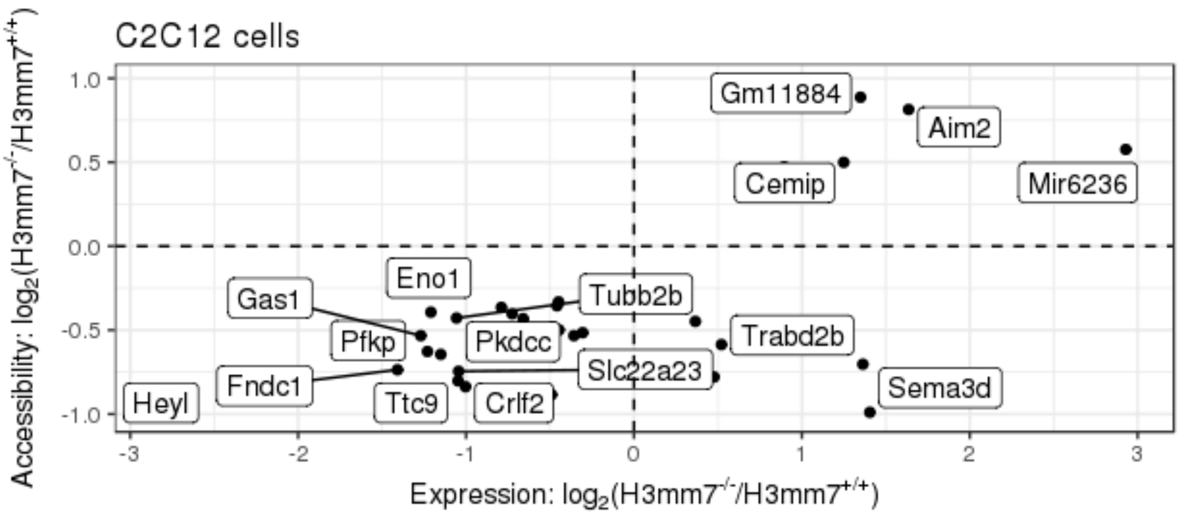

Supplementary Figure 21 | Gene expression levels were down-regulated where the chromatin accessibility of a gene promoter was decreased in H3mm7$^{-/-}$ C2C12 cells. The X-axis shows $\log_2$ fold-change of gene expression (H3mm7$^{-/-}$ to H3mm7$^{+/+}$) and the Y-axis shows $\log_2$ fold-changes of ATAC-seq read counts (H3mm7$^{-/-}$ to H3mm7$^{+/+}$) in gene promoters shown in Figure 3c.

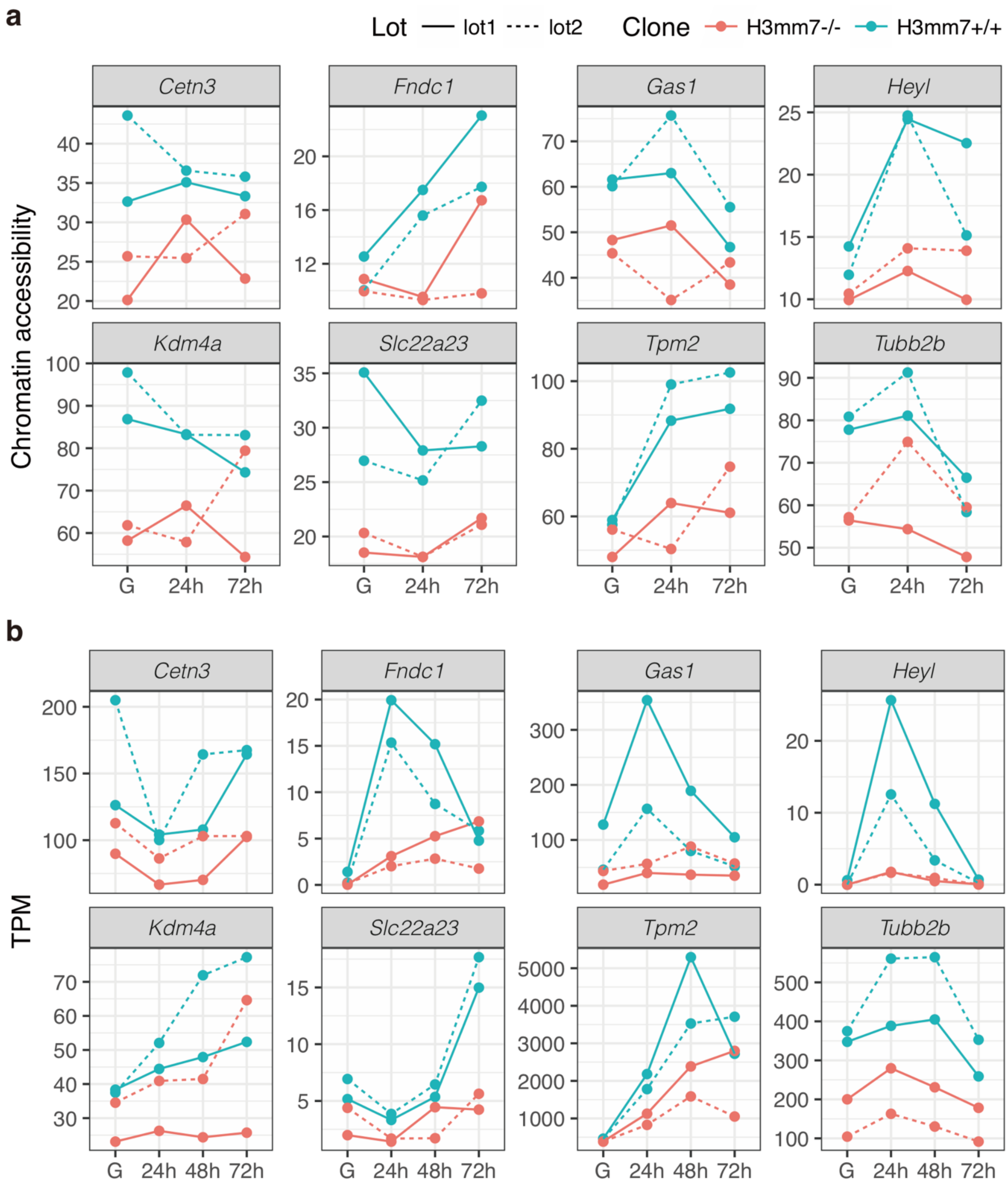

Supplementary Figure 22 | Representative genes with decreased chromatin accessibility and the corresponding expression levels in H3mm7$^{-/-}$ C2C12 cells. **(a)** Y-axis shows chromatin accessibility levels of gene promoter regions, and **(b)** the corresponding expression levels (TPM; transcripts per million) of the genes over the differentiation time course of C2C12 cells.

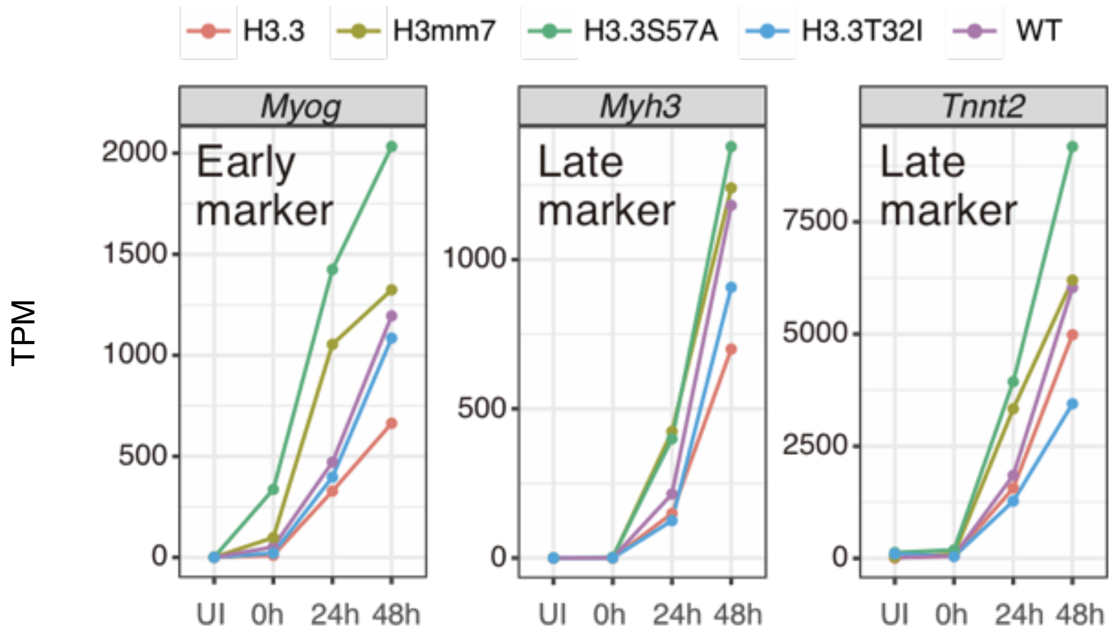

Supplementary Figure 23 | The ectopic expression of H3.3S57A enhanced the expression level of myogenic genes upon differentiation. The expression level (TPM) of representative differentiation marker genes, *Myog* (early marker), *Myh3* (late) and *Tnnt2* (late) are shown.

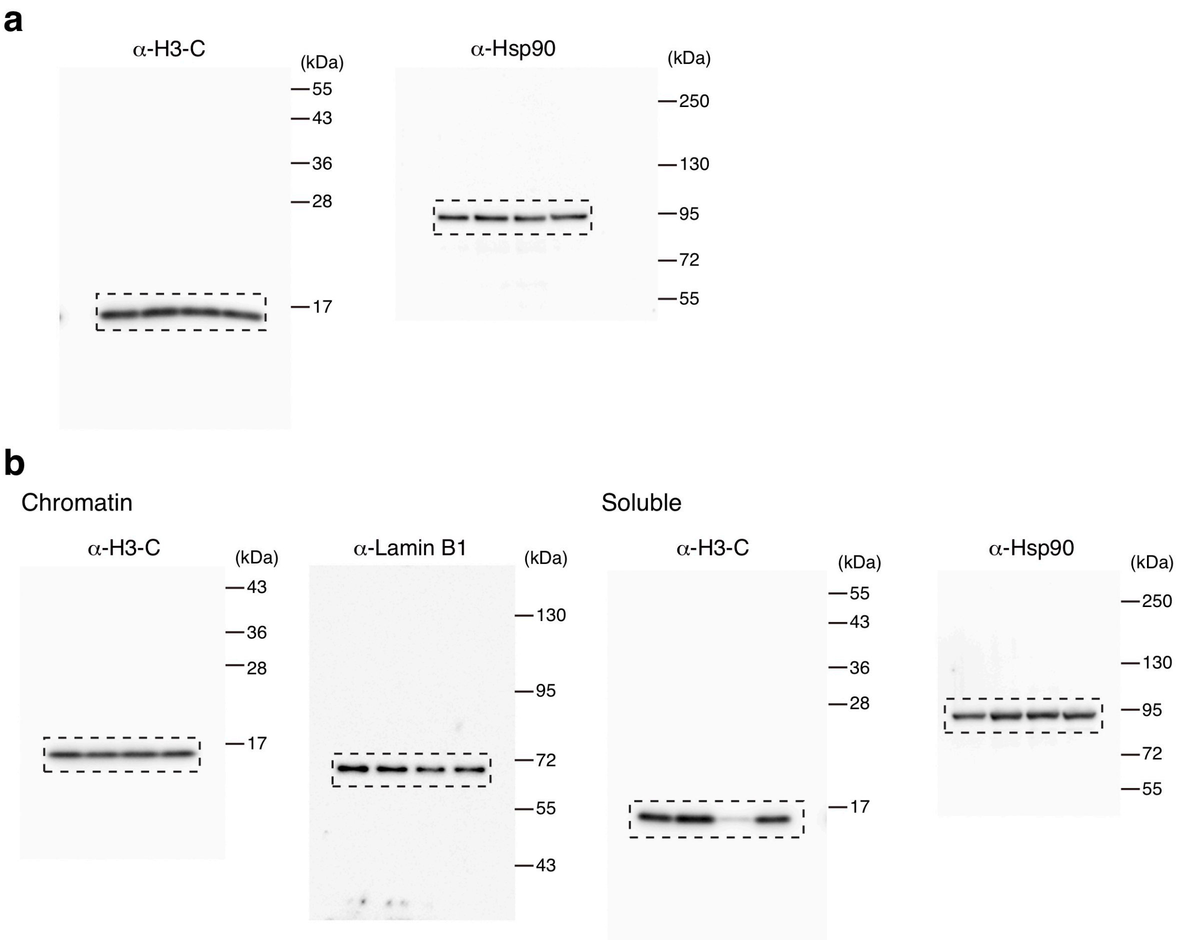

Supplementary Figure 24 | Uncropped images of immunoblotting. The dotted boxes indicate the cropped regions presented in Supplementary Figure 7.

Supplementary Table 1 | The expression levels of satellite cell marker genes in *H3mm7*$^{-/-}$ mice were comparable to those in *H3mm7*$^{+/+}$ mice at day 5 and 14 after CTX injury. The numbers show the $\log_2$ fold-changes at each comparison. The blank cells (with "-") indicate that the fold-changes of the genes did not pass the statistical test (adjusted $p > 0.3$). The underlined genes are from the gene set from Figure 1a. The fitted model in DESeq2 is: *Expression level = Intercept + Genotype effect (H3mm7*$^{+/+}$ *or* $^{-/-}$*) + Injury effect (+ or -) + Day effect (5 or 14) + Day & Injury effect (interaction)*.

Ensembl Gene ID	Gene name	Intercept	$H3mm7^{-/-}/H3mm7^{+/+}$	Injured/Uninjured	Day14/Day5	Injured & Day14
ENSMUSG00000052155	*Acvr2a*	4.22	-	-	-	-
ENSMUSG00000007655	*Cav1*	1.92	-	-	-	-
ENSMUSG00000016494	*Cd34*	1.37	-	-	-	-
ENSMUSG00000031962	*Cdh15*	1.01	-	2.18	-	-2.25
ENSMUSG00000061353	*Cxcl12*	2.65	-	-	-1.19	-
ENSMUSG00000045382	*Cxcr4*	-1.42	-	2.54	-	-
ENSMUSG00000026208	*Des*	7.78	-	-	-0.71	-
ENSMUSG00000020122	*Egfr*	-1.55	-	-	-	-
ENSMUSG00000056493	*Foxk1*	-1.48	-	-	-	-
ENSMUSG00000022484	*Hoxc10*	3.47	-	-0.81	-	-
ENSMUSG00000027009	*Itga4*	-	-	1.31	-	-
ENSMUSG00000025348	*Itga7*	3.46	-	1.58	-	-1.22
ENSMUSG00000025809	*Itgb1*	2.10	-	1.30	-	-
ENSMUSG00000025216	*Lbx1*	2.77	-	-1.28	-	-
ENSMUSG00000009376	*Met*	0.81	-	1.14	-	-
ENSMUSG00000009471	*Myod1*	1.36	-	0.92	-	-2.57
ENSMUSG00000039542	*Ncam1*	-1.56	-	4.19	-	-4.16
ENSMUSG00000000120	*Ngfr*	-	-	2.15	-	-
ENSMUSG00000028736	*Pax7*	-1.58	-	3.19	-	-
ENSMUSG00000025743	*Sdc3*	-	-	2.70	-	-
ENSMUSG00000017009	*Sdc4*	1.14	-	-	-	-
ENSMUSG00000000567	*Sox9*	1.34	-	2.84	1.78	-3.05
ENSMUSG00000027962	*Vcam1*	-	-	2.63	-	-
ENSMUSG00000032418	*Me1*	3.52	0.84	-2.70	0.75	1.72
ENSMUSG00000008958	*Vps72*	3.59	0.77	-	0.93	-
ENSMUSG00000054034	*Tceal5*	1.92	0.76	1.67	-	-1.29
ENSMUSG00000004980	*Hnrnpa2b1*	3.26	0.69	-	-	-
ENSMUSG00000107002	*0610012G03Rik*	3.46	0.68	-0.79	-	-
ENSMUSG00000019970	*Sgk1*	4.47	0.66	-1.27	-	1.03
ENSMUSG00000008855	*Hdac5*	3.05	0.65	-	-	-
ENSMUSG00000022635	*Zcrb1*	3.97	0.62	-1.70	-0.67	1.20
ENSMUSG00000034220	*Gpc1*	2.07	0.61	0.72	1.52	-1.07
ENSMUSG00000031885	*Cbfb*	3.36	0.60	-	-	-
ENSMUSG00000063856	*Gpx1*	1.99	0.59	1.82	-	-
ENSMUSG00000038733	*Wdr26*	4.45	0.54	0.50	0.58	-0.58
ENSMUSG00000026150	*Mff*	5.30	0.53	-0.46	-	-
ENSMUSG00000025351	*Cd63*	3.61	0.53	2.07	0.71	-1.73
ENSMUSG00000001506	*Col1a1*	2.81	0.53	4.00	1.64	-2.95
ENSMUSG00000030352	*Tspan9*	5.11	0.47	0.62	-	-
ENSMUSG00000025393	*Atp5b*	5.10	0.36	-0.50	-	0.56
ENSMUSG00000017300	*Tnnc2*	8.30	-0.37	-1.10	-0.54	0.92
ENSMUSG00000030647	*Ndufc2*	5.06	-0.54	-0.71	-0.73	-
ENSMUSG00000008575	*Nfib*	4.18	-0.54	-	-	-
ENSMUSG00000052305	*Hbb-bs*	6.10	-0.55	-2.14	-0.87	2.02
ENSMUSG00000024949	*Sf1*	3.44	-0.60	0.88	-	-1.04
ENSMUSG00000055322	*Tns1*	4.50	-0.60	-	-	-
ENSMUSG00000038239	*Hrc*	3.68	-0.65	-0.80	-	-
ENSMUSG00000027333	*Smox*	5.35	-0.68	-3.36	-	2.37
ENSMUSG00000053898	*Ech1*	4.02	-0.72	-1.16	-	1.27
ENSMUSG00000068614	*Actc1*	6.66	-0.72	3.48	1.86	-3.12
ENSMUSG00000042747	*Krtcap2*	3.59	-0.73	-	-1.53	-
ENSMUSG00000030433	*Sbk2*	4.18	-0.93	-1.35	-0.98	2.06

Supplementary Table 2 | Full results of GSEA analysis for DEGs using the $\log_2$ fold-change values of *H3mm7*$^{-/-}$ vs *H3mm7*$^{+/+}$ in C2C12 cells.

ID	Description	setSize	enrichmentScore	NES	pvalue	p.adjust
**GO:0042254**	ribosome biogenesis	96	-0.463	-2.666	1.53E-04	0.022
**GO:0042255**	ribosome assembly	24	-0.663	-2.657	1.71E-04	0.022
**GO:0006364**	rRNA processing	73	-0.487	-2.645	1.57E-04	0.022
**GO:0006412**	translation	178	-0.406	-2.631	1.41E-04	0.022
**GO:0043043**	peptide biosynthetic process	180	-0.400	-2.590	1.41E-04	0.022
**GO:0016072**	rRNA metabolic process	74	-0.474	-2.581	1.57E-04	0.022
**GO:0043604**	amide biosynthetic process	188	-0.392	-2.566	1.40E-04	0.022
**GO:0000028**	ribosomal small subunit assembly	14	-0.757	-2.555	1.76E-04	0.022
**GO:0042274**	ribosomal small subunit biogenesis	29	-0.599	-2.553	1.68E-04	0.022
**GO:0042273**	ribosomal large subunit biogenesis	32	-0.569	-2.500	1.67E-04	0.022
**GO:0034470**	ncRNA processing	114	-0.418	-2.486	1.50E-04	0.022
**GO:1901566**	organonitrogen compound biosynthetic process	275	-0.351	-2.441	1.32E-04	0.022
**GO:0006518**	peptide metabolic process	200	-0.365	-2.412	1.39E-04	0.022
**GO:0035914**	skeletal muscle cell differentiation	21	-0.625	-2.400	1.73E-04	0.022
**GO:0010830**	regulation of myotube differentiation	11	-0.764	-2.372	1.79E-04	0.022
**GO:0034660**	ncRNA metabolic process	137	-0.381	-2.354	1.46E-04	0.022
**GO:0043603**	cellular amide metabolic process	215	-0.349	-2.333	1.38E-04	0.022
**GO:0014902**	myotube differentiation	25	-0.547	-2.219	3.43E-04	0.024
**GO:0000470**	maturation of LSU-rRNA	15	-0.633	-2.187	1.76E-04	0.022
**GO:0002181**	cytoplasmic translation	20	-0.576	-2.181	1.04E-03	0.042
**GO:0022613**	ribonucleoprotein complex biogenesis	140	-0.350	-2.171	1.46E-04	0.022
**GO:1901564**	organonitrogen compound metabolic process	383	-0.299	-2.170	1.28E-04	0.022
**GO:0046939**	nucleotide phosphorylation	19	-0.575	-2.144	1.04E-03	0.042
**GO:0006165**	nucleoside diphosphate phosphorylation	18	-0.583	-2.134	1.04E-03	0.042
**GO:0007519**	skeletal muscle tissue development	44	-0.443	-2.114	4.92E-04	0.027
**GO:0060538**	skeletal muscle organ development	44	-0.443	-2.114	4.92E-04	0.027
**GO:0006457**	protein folding	54	-0.396	-1.996	9.72E-04	0.042
**GO:0006396**	RNA processing	262	-0.258	-1.782	2.67E-04	0.022
**GO:0002682**	regulation of immune system process	185	0.226	1.673	1.05E-03	0.042
**GO:0016477**	cell migration	227	0.218	1.677	1.11E-03	0.044
**GO:0030030**	cell projection organization	286	0.210	1.678	8.14E-04	0.040
**GO:0006928**	movement of cell or subcellular component	308	0.210	1.702	4.16E-04	0.025
**GO:0051270**	regulation of cellular component movement	161	0.242	1.740	1.33E-03	0.048
**GO:0006952**	defense response	163	0.245	1.766	1.00E-03	0.042
**GO:0040012**	regulation of locomotion	152	0.250	1.774	1.33E-03	0.048
**GO:2000145**	regulation of cell motility	147	0.252	1.784	9.72E-04	0.042
**GO:0009653**	anatomical structure morphogenesis	494	0.210	1.794	5.52E-04	0.030
**GO:0048646**	anatomical structure formation involved in morphogenesis	234	0.233	1.797	3.79E-04	0.025
**GO:0007167**	enzyme linked receptor protein signaling pathway	163	0.250	1.804	6.67E-04	0.035
**GO:0040011**	locomotion	263	0.230	1.806	4.00E-04	0.025
**GO:0030334**	regulation of cell migration	142	0.260	1.818	9.67E-04	0.042
**GO:0032989**	cellular component morphogenesis	260	0.237	1.856	3.98E-04	0.025
**GO:0001568**	blood vessel development	118	0.281	1.894	9.19E-04	0.042
**GO:0000902**	cell morphogenesis	240	0.248	1.927	3.81E-04	0.025

**GO:0048514**	blood vessel morphogenesis	96	0.299	1.932	1.15E-03	0.044
**GO:0001654**	eye development	80	0.320	1.977	5.60E-04	0.030
**GO:0030031**	cell projection assembly	95	0.308	1.989	8.61E-04	0.042
**GO:0050808**	synapse organization	45	0.386	2.035	1.28E-03	0.048
**GO:0032990**	cell part morphogenesis	182	0.277	2.042	3.46E-04	0.024
**GO:0002252**	immune effector process	106	0.316	2.088	3.00E-04	0.023
**GO:0017015**	regulation of transforming growth factor beta receptor signaling pathway	23	0.491	2.095	9.50E-04	0.042
**GO:1903844**	regulation of cellular response to transforming growth factor beta stimulus	23	0.491	2.095	9.50E-04	0.042
**GO:0098792**	xenophagy	23	0.504	2.153	4.75E-04	0.027
**GO:0048858**	cell projection morphogenesis	173	0.297	2.163	3.43E-04	0.024
**GO:0050691**	regulation of defense response to virus by host	27	0.487	2.194	2.45E-04	0.022
**GO:0090288**	negative regulation of cellular response to growth factor stimulus	27	0.489	2.201	2.45E-04	0.022
**GO:1901343**	negative regulation of vasculature development	18	0.562	2.201	1.18E-03	0.045
**GO:0051928**	positive regulation of calcium ion transport	19	0.552	2.206	7.07E-04	0.036
**GO:0030512**	negative regulation of transforming growth factor beta receptor signaling pathway	16	0.595	2.233	2.33E-04	0.022
**GO:1903845**	negative regulation of cellular response to transforming growth factor beta stimulus	16	0.595	2.233	2.33E-04	0.022
**GO:0010927**	cellular component assembly involved in morphogenesis	73	0.370	2.238	2.74E-04	0.022
**GO:0050688**	regulation of defense response to virus	29	0.490	2.259	4.92E-04	0.027
**GO:0002230**	positive regulation of defense response to virus by host	24	0.528	2.289	2.40E-04	0.022
**GO:0044782**	cilium organization	65	0.392	2.304	2.69E-04	0.022
**GO:0060271**	cilium morphogenesis	65	0.397	2.333	2.69E-04	0.022
**GO:0042384**	cilium assembly	57	0.434	2.446	2.66E-04	0.022
**GO:0048536**	spleen development	11	0.780	2.562	2.26E-04	0.022

Preranked GSEA analysis for gene ontology terms of biological processes was performed.

Supplementary Table 3 | Results of GSEA analysis for DEGs using the log_2 fold-change values of H3mm7+ vs the control in NIH3T3 cells.

ID	Description	setSize	enrichmentScore	NES	pvalue	p.adjust
**GO:0007586**	digestion	13	0.783	2.425	1.92E-04	0.068
**GO:0022600**	digestive system process	12	0.764	2.312	1.93E-04	0.068
**GO:0019233**	sensory perception of pain	15	0.695	2.242	3.80E-04	0.104
**GO:0032943**	mononuclear cell proliferation	47	0.503	2.238	1.73E-04	0.068
**GO:0042493**	response to drug	49	0.495	2.228	1.71E-04	0.068
**GO:0070661**	leukocyte proliferation	48	0.494	2.211	1.72E-04	0.068
**GO:0046651**	lymphocyte proliferation	45	0.495	2.183	1.73E-04	0.068
**GO:0006937**	regulation of muscle contraction	30	0.545	2.166	5.38E-04	0.133
**GO:0006941**	striated muscle contraction	28	0.540	2.105	1.26E-03	0.223
**GO:0044057**	regulation of system process	79	0.415	2.074	3.28E-04	0.101
**GO:1901861**	regulation of muscle tissue development	27	0.537	2.070	1.44E-03	0.238
**GO:0032944**	regulation of mononuclear cell proliferation	35	0.488	2.011	1.07E-03	0.204
**GO:0003008**	system process	229	0.327	1.990	1.41E-04	0.068
**GO:0007155**	cell adhesion	284	0.266	1.672	8.23E-04	0.185
**GO:0022610**	biological adhesion	286	0.263	1.652	9.59E-04	0.198

Preranked GSEA analysis against gene ontology terms of biological processes was performed.

Supplementary Table 4 | gRNA and primer list for H3mm7 locus deletion

H3mm7-upstream-1st	TATAGATAAAGGAATAACTACGG
H3mm7-downstream-1st	ACATCACTTATCTATTGCTTTGG
H3mm7-upstream-2nd	GGATATGTTGGTTAGCGGAAAGG
H3mm7-downstream-2nd	AGGTCTCATCTTTGGGCTGATGG
H3mm7-upstream-3rd	CCTTCGCTCTCCAACGCCAGCGC
H3mm7-downpstream-3rd	TCGTAAGTAGATATTGATATAGG
H3mm7 genotyping primer Fw	TGTTATTACCCCTGGATAAAAGCCGGAAAG
H3mm7 genotyping primer Rv	CATAATTGGATTTGGAAAAGTTTTGGCGGC

Supplementary Table 5 | Quantitative RT-PCR primer list

Des	5′-TTTCTCCACTCACAGGCTCTGACC-3′
	5′-GAGCTGGGTTCTCTCTTAAGAGCC-3′
Ckm	5′-AAGTCCAATCATTGGGCTCTGTCC-3′
	5′-ACGGACTTTTATTTAAGGCAGGGC-3′
Gapdh	5′-GGTTTCTTACTCCTTGGAGGCCAT-3′
	5′-GGTTTCTTACTCCTTGGAGGCCAT-3′
Eef1a1	5′-CTCTGACTACCCTCCACTTGGTCG-3′
	5′-ATTAAGACTGGGGTGGCAGGTGTT-3′
Myh3	5′-AAAAGGCCATCACTGACGC-3′
	5′-CAGCTCTCTGATCCGTGTCTC-3′
Ryr1<br>primer bank ID: 30387855a1	5′-CAGTTTTTGCGGACGGATGAT-3′
	5′-CACCGGCCTCCACAGTATTG-3′
Svil<br>primer bank ID: 23346601a1	5′-GTCCCAAAGAGACATTCGAGAAA-3′
	5′-CTGTGTGTGTGAACGGTCCT-3′
Csrp3	5′-CAAGAGTCCCCTTACTCAGTTG-3′
	5′-ACTGGATTTCTTCTGCATGGTAG-3′
Klhl41	5′-CAGAAACCATTCCAGTCTTGTTAC-3′
	5′-CACTCAGATGTTACGTTATCAAGC-3′
Acta1	5′-TTGTGCACCGCAAATGCTTCTAGG-3′
	5′-ATGTACACGTCAAAAACAGGCGCC-3′
Tnni2	5′-GAGAATCTGAGAAGGAGAACTACC-3′
	5′-CCTTCACCTCCATGTCATATTTCT-3′
Tnnt2	5′-CAGAGGAGGCCAACGTAGAAG-3′
	5′-CTCCATCGGGGATCTTGGGT-3′

Supplementary Table 6 | Variant-specific quantitative RT-PCR primer list

H3mm7 Forward primer	5′-TCAGACGCTATCAGAAGG-3′
H3f3a Forward primer	5′-CCGTGAAATCAGACGCTATCAGAAGT-3′
Blocker oligo	5′-GCTATCAGAAGTCCACTGAACTT-3′
Reverse primer	5′-CCAGACGCTGAAAGGG-3′

Supplementary Table 7 | Data collection and refinement statistics

	mouse nucleosome containing H2A, H2B type3-A, H3.3, and H4	mouse nucleosome containing H2A, H2B type 3-A, H3mm7, and H4
Data collection		
Wavelength (Å)	0.9800	1.1000
Space group	$P2_12_12_1$	$P2_12_12_1$
Cell dimensions		
a, b, c (Å)	a = 106.523<br>b = 110.095<br>c = 182.264	a = 105.550<br>b = 109.380<br>c = 176.209
α, β, γ (°)	α = β = γ = 90	α = β = γ = 90
Resolution (Å)	50.00 - 2.87 (2.97 - 2.87)	50.00 - 3.45 (3.57 - 3.45)
Reflections (Unique)	49349	28747
R_{merge} (%)	8.2 (50.0)	9.7 (42.6)
Mean $<I/\sigma(I)>$	21.9 (3.3)	19.7 (3.2)
Completeness (%)	99.6 (100.0)	99.3 (98.9)
Redundancy	4.2 (4.1)	5.9 (5.8)
Refinement		
Resolution (Å)	48.904 - 2.874	34.875 - 3.450
$R_{work/free}$ (%)	21.89 / 25.53	19.97 / 25.44
R.m.s deviations		
bond length (Å)	0.010	0.011
bond angles (°)	1.194	1.247
B factors(Å^2)		
Protein	44.62	77.87
DNA	97.75	146.36
Solvent	41.32	-
Ramachandran plot		
residues in favorable resions (%)	97.41	96.19
residues in allowed regions (%)	2.59	3.81
PDB ID	5XM0	5XM1

Values in parentheses are for the highest-resolution shell.

$R_{merge} = \Sigma_{hkl} \Sigma_i \mid I_i(hkl) - \langle I(hkl) \rangle \mid / \Sigma_{hkl} \Sigma_i I_i(hkl)$, where $Ii\,(hkl)$ is the intensity of an observation and $\langle I(hkl) \rangle$ is the mean value for that reflection.

$R_{work} = \Sigma_{hkl} \; ||F_O| - |F_C|| / \Sigma_{hkl} |F_O|$, where Fo and Fc are the observed and calculated structure-factor amplitudes, respectively.

R_{free} was calculated with 5% of the data excluded from the refinement.
